# Fiber Optic LSPR Sensing AFM1 in Milk with Enhanced Sensitivity by the Hot Spot Effect Based on Nanogap Construction

**DOI:** 10.3390/mi15060779

**Published:** 2024-06-13

**Authors:** Jiacong Li, Yuxin Ni, Wei Zhang, Elvige Laure Nteppe Nteppe, Yurong Li, Yeshun Zhang, Hui Yan

**Affiliations:** School of Biotechnology, Jiangsu University of Science and Technology, Zhenjiang 212100, China; li1782372540@163.com (J.L.); 212211801113@stu.just.edu.cn (Y.N.); 211111802124@stu.just.edu.cn (W.Z.); elvigenteppe@gmail.com (E.L.N.N.); yrli@just.edu.cn (Y.L.); zyssri@just.edu.cn (Y.Z.)

**Keywords:** localized surface plasmon resonance (LSPR), fiber optical, mycotoxins, aflatoxin M1, hot spots

## Abstract

The detection of the amount of aflatoxin M1 (AFM1) in milk is crucial for food safety. Here, we utilize a fiber optic (FO) localized surface plasmon resonance (LSPR) biosensor by constructing gold nanoparticle (AuNP) multimers, in which the nanogaps amplified the LSPR signal by the hot spot effect, and achieved a highly sensitive detection of f AFM1. Through the optimization of parameter conditions for the fabrication of the sensor and detection system, a high performance result from the FO LSPR biosensor was obtained, and the method for AFM1 detection was established, with a wide detection range of 0.05–100 ng/mL and a low limit of detection (LOD) of 0.04 ng/mL, and it has been successfully validated with the actual sample milk. Therefore, it is a good strategy to fabricate highly sensitive FO LSPR sensors for detecting AFM1 by constructing AuNP multimers, and this approach is suitable for developing other biosensors.

## 1. Introduction

Aflatoxins (AFs) are a group of toxic metabolites including AFB1, AFG1, AFG2, and AFM1 produced by *Aspergillus flavus* and *Aspergillus parasiticus*, which easily grow on grains, feeds, and nuts [1,2]. Due to AF contamination, corn farmers in the United States lose USD 1.6 billion annually, while losses in sub–Saharan Africa reach USD 4.5 billion; overall, 38% of global agricultural losses are caused by AFs [3].

Among the AF family, AFM1, one of the most common mycotoxins, poses a great threat to human health [4]. After livestock consume AFB1-contaminated feed, AFB1 is metabolized in the liver to generate the hydroxyl derivative AFM1, and then migrates to meat, milk, eggs, etc., of which milk is the most common [5,6].

AFM1 is carcinogenic, teratogenic, mutagenic, genotoxic, and immunotoxic [7]. The most important damage that AFM1 causes to the human body is its toxicity to the liver [8]. It has been reported that there is a quantitative relationship between the level of AFM1 and the risk of hepatocellular carcinoma in people carrying the hepatitis B virus [9]. Furthermore, AFM1 can disrupt the gut and has an intestinal immunosuppressive effect [10]. Therefore, the International Agency for Research on Cancer (IARC) classifies AFM1 as a group 2B carcinogen (probably carcinogenic to humans) [11]. Many countries have set a maximum allowable level of AFM1 in milk; the US Food and Drug Administration (FDA) stipulates 500 ng/kg and the EU stipulates 50 ng/kg [12].

The most effective way to avoid the harm of AFM1 is to detect it in time. The traditional methods for the detection of AFM1 are high performance liquid chromatography (HPLC) [13], liquid chromatography coupled to tandem mass spectrometry (LC-MS/MS) [14], enzyme-linked immunosorbent assay (ELISA) [15], and thin layer chromatography (TLC) [16], and they each have disadvantages, such as complex operation, expensive equipment, high technical requirements for testing personnel, and difficulty being implement in professional laboratories. To tackle these problems, sensing technology is a good choice.

Currently, the biosensors for the detection of AFM1 attracted much attention. Using DNA fragments that can specifically capture AFM1, Dinckaya et al. developed a biosensor, and the detection range was ~1–14 ng/mL [17]. Based on fluorescence resonance energy transfer (FRET), Li et al. constructed a AFM1 sensor with a detection range of ~5–150 pg/mL and a LOD of 1.5 pg/mL, presenting high sensitivity [18].

In regard to sensors, localized surface plasmon resonance (LSPR) is often used. LSPR is an optical phenomenon that occurs when a light wave is trapped by precious metal nanoparticles (NPs) smaller than its wavelength, which is the result of the interaction between the incident light and the electrons on the surface of NPs. Precious metal nanostructures can absorb photon energy and present significant extinction and near-field enhancement characteristics [19]. When LSPR is excited, a peak appears in the extinction spectrum of the noble metal NPs, and the wavelength of the peak is called the resonance wavelength (λmax), and the position of the peak is extremely sensitive to the refractive index (RI) of the environment near the surface of the noble metal NPs [20]. Therefore, when molecules are bound to noble metal NPs, the RI around the NPs changes, resulting in the shift of the resonance absorption peak [21]. Based on this principle, LSPR can be applied for sensing in food safety [22], environmental monitoring [23], and biomedicine [24].

Fiber optic (FO) LSPR sensors combine fiber optics with LSPR, and they have the advantages of miniaturization, low cost, easy preparation, and real-time detection [25,26,27]; many up-to-date FO LSPR sensors have been reported. Jeong et al. [27] prepared a FO LSPR sensor which successfully detected prostate-specific antigen (PSA) by assembling the AuNPs on the fiber end face, and obtained a LOD of 1 pg/mL. In 2020, Barroso et al. [28] fabricated a FO LSPR sensor for Cu^2+^ detection, with a detection range of 10^−12^~10^−4^ M and a LOD of 0.2 PM.

Because of the limited amount of sensing material on FO, the sensitivity of FO LSPR sensors needs to be improved for the detection of lower concentration analytes, and how to improve the sensitivity is still a challenge. Currently, some strategies have been tried. 

Luo et al. [29] developed a U-shaped FO LSPR sensor to increase the sensing area for the cancer cell detection, with a wide detection range of 1 × 10^2^~1 × 10^6^ cells/mL and a LOD of 30 cells/mL, which was more than 29 times higher than that of the straight sensor. Xu et al. [30] proposed a Ω-shaped FO LSPR sensor, whose sensitivity was 2.5 times higher than the U-type sensor. Nevertheless, because the straight FO has the advantages of being easy to fabricate and use, improving its sensitivity attracts researchers’ interest.

Hot spots are a phenomenon wherein the electromagnetic field strength is amplified by two to five orders in the nanogap between precious metals NPs, and molecular sensing by hot spots has a high sensitivity [19,31,32]. Therefore, it is possible to improve the sensitivity of the sensors by constructing hot spots. Kim et al. [33] constructed AuNP dimers to enhance the electromagnetic field strength, resulting in 9.1 times higher sensitivity than that of the monomer FO LSPR sensor. On the fiber end face, we successfully fabricated the AuNP multimers through which the nanogaps are created between AuNPs, and the sensitivity of the deoxynivalenol sensor was increased by eight times, when compared with that of an ordinary monomer FO LSPR sensor [21].

**Scheme 1:**
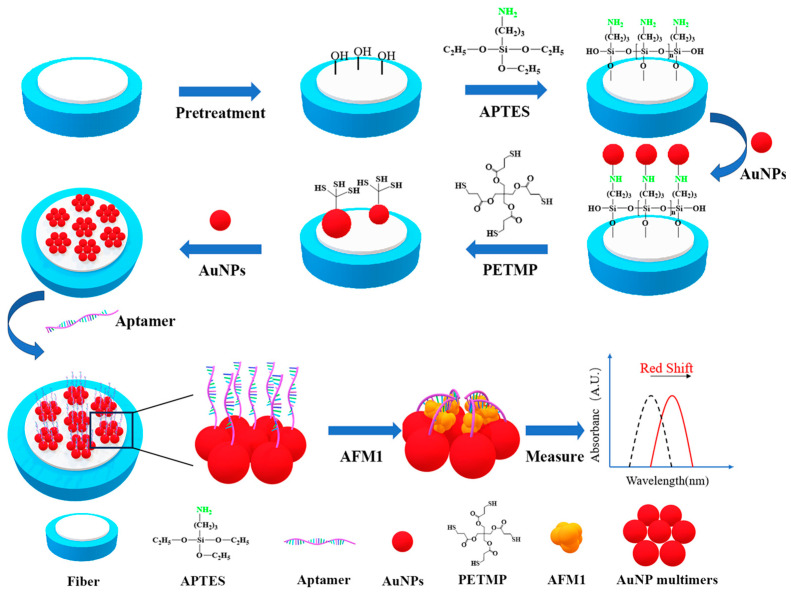
Principle and preparation process of the FO LSPR biosensor for AFM1 detection.

In view of the above, a FO LSPR sensor for AFM1 based on the AuNP multimers was constructed in this study to achieve the on-site and highly sensitive detection of AFM1. As shown in Scheme 1, the FO end face was treated with a piranha solution to generate hydroxyl groups, and 3-aminopropyltriethoxysilane was coupled to generate NH_2_ groups on the fiber end face. The first batch of AuNPs was anchored through Au-NH_2_ bond, and then was treated with PETMP. The second batch of AuNPs was bound through Au-S bonds, generating the AuNP multimers. Subsequently, AFM1 aptamer was attached to the AuNPs. The aptamer specifically captured AFM1, leading to the change in refractive index of the environment around the AuNPs, then the LSPR peak shifted, through which the concentration of AFM1 could be determined. The peak shift signal was amplified by the hot spots effect, so the lower content of AFM1 could be detected.

## 2. Materials and Methods

### 2.1. Materials

Tris(hydroxymethyl)aminomethane hydrochloride (Tris-HCL), gold chloride trihydrate, 3-aminopropyltriethoxysilane (APTES) were supplied by Shanghai Aladdin Biochemical Science and Technology Co., Ltd. (Shanghai, China). Tetrakis (3-mercaptopropionic acid) pentaerythritol ester (PETMP), AFM1, and aflatoxin B1 (AFB1) were purchased from Shanghai Yien Chemical Technology Co., Ltd. (Shanghai, China). Trisodium citrate dihydrate was purchased from Sinopharm Holding Chemical Reagent Co., Ltd. (Shanghai, China). Sulfhydryl-modified AFM1 aptamer sequence 5‘-(SH)-(CH_2_)_6_—ACT GCT AGA GAT TTT CCA CAT-3′ [34] was synthesized by Shanghai Sangon Biotech Co., Ltd. (Shanghai, China). AFM1 ELISA kit was purchased from Shanghai Yulong Biotechnology Co., Ltd. (Shanghai, China). Milk sample was purchased from a local supermarket. All other chemicals were analytically pure, and ultrapure water was used in this work.

### 2.2. LSPR Spectrum Collection System

A high throughput multimode bare FO was utilized to fabricate the biosensor, and a self-made spectrum collection system was applied in this work [21]. One of the ends of FO was used as the sensing surface and another end was connected to the Y-shaped FO adapter, containing two long branches, one was a single FO receiving the light from a tungsten halogen lamp and the other contained three FO in a linear arrangement, which transferred the LSPR spectra to a CCD spectrometer.

### 2.3. Methods

#### 2.3.1. Simulation of the Hot Spots in the AuNP Multimers

To investigate the enhancement of the local electromagnetic field by the AuNP multimers, a finite-difference time-domain (FDTD) simulation was applied using a full-field scattering source; the wavelength was 520 nm and the direction was along the z-axis, in addition, the refractive index of the surrounding medium was 1.33, through which the electromagnetic interaction and the field enhancement effect produced by the AuNP multimers could be simulated.

#### 2.3.2. Synthesis of the AuNPs

The AuNPs were synthesized according to the Frens method [35] with slight modifications. Briefly, 100 mL 0.01% (*w*/*w*) of gold chlorate was added to a 250 mL three-necked flask and heated to boiling under reflux and stirring. Then, 3 mL of 1.0% (*w*/*w*) sodium citrate solution was rapidly dripped to the solution and heated for 15 min. The solution was then cooled to room temperature, collected, and stored at 4 °C.

#### 2.3.3. Pretreatment of the FO End Face

One of the FO end faces was immersed in aqua regia (concentrated hydrochloric acid–concentrated nitric acid = 3:1, *v*/*v*) for 30 min; then, it was processed in a piranha solution (concentrated sulfuric acid–30% hydrogen peroxide = 7:3, *v*/*v*) at 80 °C for 40 min to remove organic matter and generate hydroxyl, and then cleaned by ultrasound with ultrapure water for 5 min, dried with N_2_.

#### 2.3.4. Amination of the FO End Face

The hydroxylated FO end face was immersed in a APTES solution (APTES:C_2_H_5_OH:H_2_O = 1:1:98, *v*/*v*/*v*) at 25 °C for 12 h, and then rinsed with ultrapure water and dried with N_2_. In this process, APTES was hydrolyzed to form highly reactive silanol, which polymerized with other silanol to form an organosilane network on the FO end face, and completed the process of amination.

#### 2.3.5. Preparation of the AuNP Multimers

The aminated FO was treated by immersing it in the AuNP dispersion at 25 °C for 5 h; meanwhile, the AuNPs were connected to the FO end face by Au-NH_2_ bond, and rinsed with ultrapure water and dried with N2. The AuNP coupled end face was immersed in 4 mg/mL PETMP (ethanol as solvent) at 25 °C for 3 h. PETMP contained four sulfhydryl groups, some of which were connected to AuNPs via Au-S bonds, and the rest sulfhydryl groups were exposed on the surface of AuNPs. Subsequently, it was immersed in the AuNP solution again at 25 °C for 5 min; meanwhile, the free sulfhydryl groups captured AuNPs through Au-S bonds to form the AuNP-PETMP-AuNP multimers. The optimal parameters referring to in the literature were applied [21].

#### 2.3.6. Coupling AFM1 Aptamer on the AuNP Multimers

The AuNP multimers prepared on the FO end face were immersed in a AFM1 aptamer solution at 25 °C for 12 h, then repeatedly rinsed with the aptamer buffer (20 mM Tris-HCL, containing 100 mM NaCl, 5 mM KCl, 2 mM MgCl_2_, and 1 mM CaCl_2_, pH 7.4) to remove any free AFM1 aptamer. Finally, the preparation of the FO LSPR biosensor was completed.

A FO sensor costs approximately CNY 10, with a production cycle of 2 days and the biosensor can be recycled and reprocessed to reduce costs, the method is as follows: the used fiber is soaked in aqua regia for 30 min to remove the AuNP multimers, the aptamer, and AFM1. Then, the new sensor can be re-prepared by the above steps. If the FO end face was accidentally worn, it can be grinded and polished for reuse.

#### 2.3.7. Characterization of the AuNPs and the AuNP Multimers

The particle size of the AuNPs was measured using a laser particle size analyzer (ZEN3600, Malvern Panalytical, Malvern, UK). Transmission electron microscopy (TEM) imaging of the AuNPs was performed using an electron microscope (HT7800, Hitachi, Tokyo, Japan) at a vacuum pressure of 3.6 × 10^5^ Pa and an accelerating voltage of 10.0 kV. The morphology of AuNPs and AuNP multimers at the FO end face was observed using a field emission scanning electron microscope (SEM, Quanta FEG 250, FEI, Waltham, MA, USA).

#### 2.3.8. Establishment of the Method for AFM1 Detection

For the parameters of the spectrometer collecting the LSPR spectra, the integration time was set 60 ms and the scanning times was set to 32. The FO LSPR sensors were used to detect different concentrations of AFM1 solution at 37 °C for 1 h, repeatedly rinsed with the AFM1 buffer (137 mM NaCl, 2.7 mM KCl,10 mM Na_2_HPO_4_, and 2 mM K_2_HPO_4_, pH7.0) to remove the uncaptured AFM1, and then were used to collect the LSPR spectra. After establishing the working curve and obtaining the performance parameters of detection range, the limit of detection (LOD), sensitivity, and reproducibility (short term, long term), the results were compared with those of the other methods.

#### 2.3.9. Detection of the Actual Sample

In this work, milk was selected as an actual sample for the performance evaluation of the method. For the detection of AFM1 in milk, the pretreatment of samples was referred to the national standard (GB5009.24-2016) [36]. We then took about 100 g of milk and shook it well, after which 10 g of milk was centrifuged at 6000 r/min for 10 min and then measured directly. A commercial ELISA kit was used as a reference method for the comparison of the detection performance with the established method.

## 3. Results and Discussion

### 3.1. FDTD Simulation

**Figure 1:**
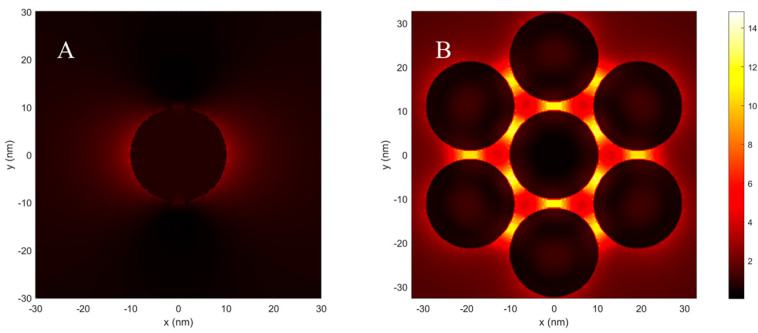
FDTD simulation: (**A**) electromagnetic field distribution of a AuNP, (**B**) electromagnetic field distribution of the AuNP multimer.

The enhancement of the electromagnetic field by the AuNPs and the AuNP multimers was simulated using the FDTD method. As shown in Figure 1A, the electromagnetic field around the AuNP was weak; however, it was very strong in the nanogaps of the AuNP multimers (Figure 1B) because the hot-spot effect occurred in the nanogaps.

### 3.2. Characterization of the AuNPs

**Figure 2:**
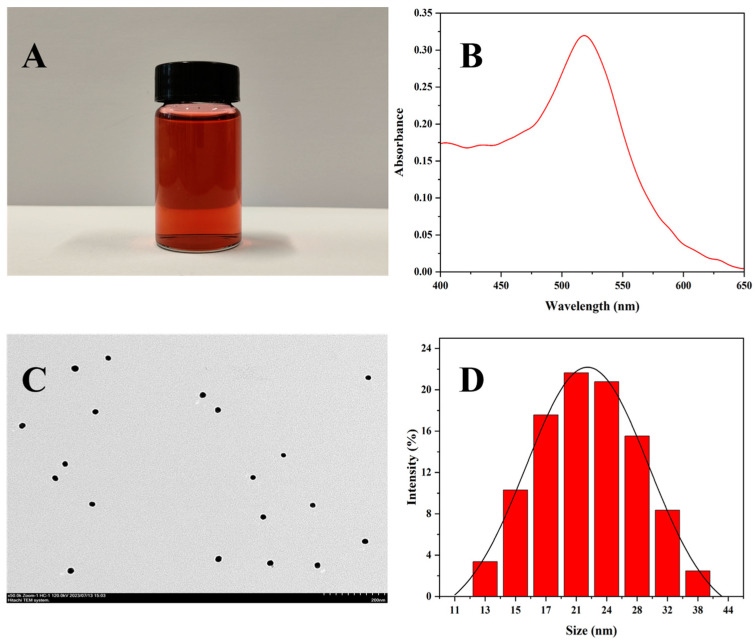
Characterization of the AuNPs: (**A**) appearance of the AuNP solution, (**B**) visible absorption spectrum of the AuNP solution, (**C**) TEM image of the AuNPs, and (**D**) size distribution of the AuNPs.

The synthesized AuNPs were wine-red (Figure 2A), and their visible absorption spectrum is shown in Figure 2B, in which the absorption peak was located at around 520 nm. The TEM image of the AuNPs is shown in Figure 2C; they appeared spherical and had good dispersion. Their particle size distribution is shown in Figure 2D; the average diameter was about 21 nm. The above data were consistent with the literature [37], which demonstrated that the AuNPs were successfully synthesized.

### 3.3. Condition Optimization

For FO LSPR biosensors, the condition parameters of the fabrication and the detection system have a great influence on the detection performance of the sensor. In this work, the AFM1 aptamer concentration for the construction of biosensor, the AFM1 capture time, and the pH of the detection system were optimized. The LSPR peak shift was used as the indicator for the performance evaluation of the biosensor during the measurement process.

**Figure 3:**
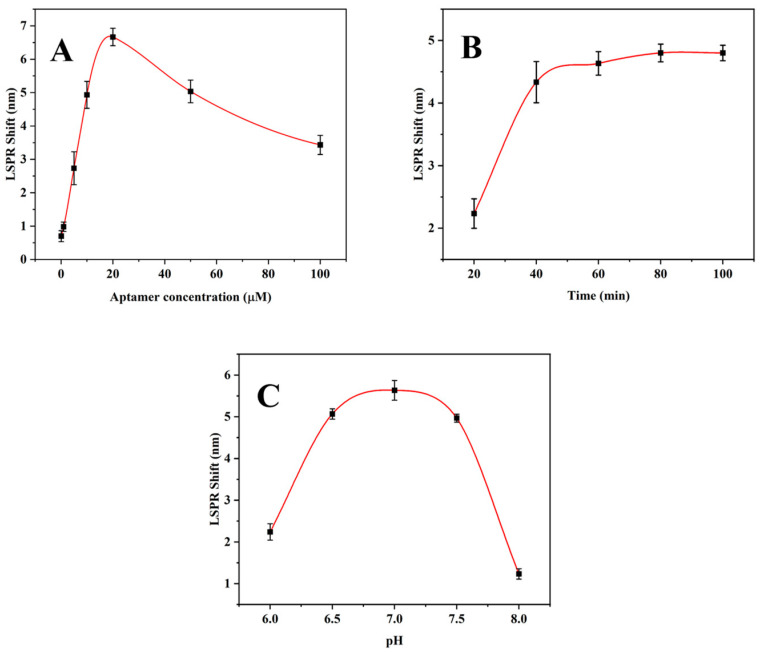
Effect of conditions on the sensor performance: (**A**) the capture time for capturing AFM1, (**B**) the pH of the detection system, and (**C**) the concentration of AFM1 aptamer used for the fabrication of the sensor.

The amount of the aptamer probe on the FO LSPR biosensor affected the sensor performance. When its concentration was low, the aptamer quantity on the end face was too small to capture enough AFM1, leading to a smaller LSPR peak shift. While the concentration of aptamer was too high, the aptamer on the end face was too much, the aptamer could not capture more AFM1 because of the spatial site resistance and electrostatic interaction between the aptamer, resulting in a small LSPR peak shift; therefore, the concentration of the aptamer was optimized. The aptamer solutions with different concentrations of 0.1, 1, 5, 10, 20, 50, and 100 μM were used to be coupled on the AuNP multimers, then 50 ng/mL AFM1 was detected. The results are shown in Figure 3A, obviously, the largest LSPR peak shift was reached at 20 μM. Therefore, a 20 μM AFM1 aptamer was chosen for the preparation of the sensor.

The capture time affected the captured quantity of AFM1 by the aptamer. In order to obtain the optimal capture time, the biosensors were used to detect the 50 ng/mL AFM1 solution, and the LSPR peak shift was recorded every 20 min in 100 min. The results are shown in Figure 3B, wherein the LSPR peak shift increased continuously in the range of 20–60 min; however, with the time extended from 60 min to 100 min, the peak shift did not increase, demonstrating that 60 min was the optimal capture time.

For the measurement of AFM1, the pH (6.0, 6.5, 7.0, 7.5, and 8.0) of detection system was optimized with 50 ng/mL, and the results are shown in Figure 3C. The LSPR peak shifts were smaller under acidic and alkaline conditions, and the maximum value was at pH 7. Therefore, the optimal pH was 7.0.

### 3.4. Detection of AFM1 with the FO LSPR Sensor

**Figure 4:**
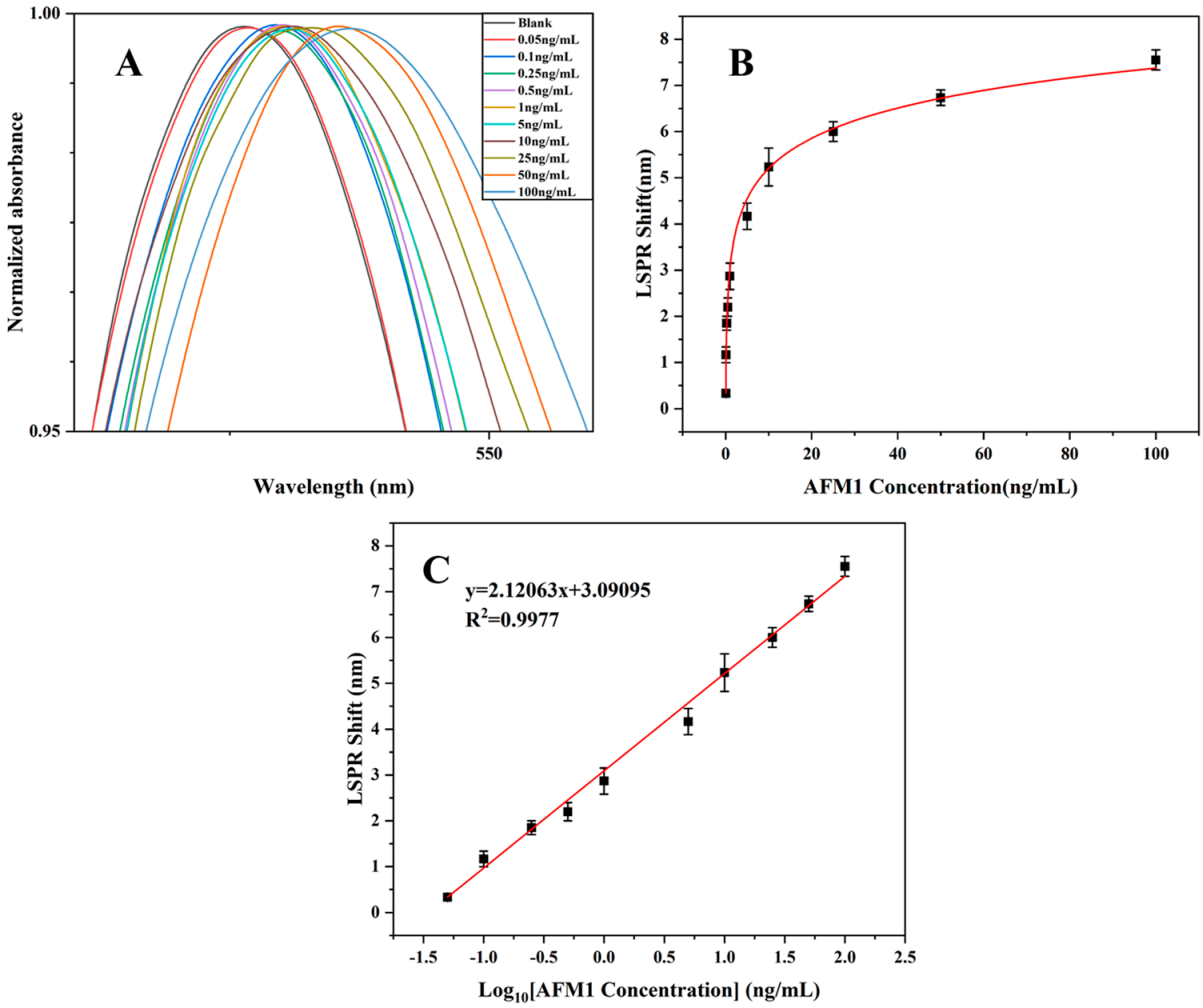
LSPR sensor response to AFM1: (**A**) the normalized LSPR spectra, (**B**) the plot of the LSPR peak shifts vs. AFM1 concentrations, and (**C**) the linear relationship between the LSPR peak shift and the logarithm concentration of AFM1.

Different concentrations of AFM1 (0.05, 0.1, 0.25, 0.5, 1, 5, 10, 25, 50, and 100 ng/mL) were measured by the FO LSPR biosensor, and different LSPR spectra were obtained. The results are shown in Figure 4A, and the plot of LSPR peak shifts vs. AFM1 concentrations are presented in Figure 4B, the shift of the LSPR peak rose with the increase in AFM1 concentration, presenting a non-linear relationship. After the transformation of logarithm, there was a linear relationship between the logarithm concentration of AFM1 and the LSPR peak shift, which was shown in Figure 4C. The fitted regression equation was y = 2.1206x + 3.0910, R^2^ was 0.9977, demonstrating the good linear relationship. The detection range was ~0.05–100.0 ng/mL and the LOD was calculated based on that the signal (S) was 3-fold of noise (N); it was 0.04 ng/mL (S/N = 3).

The detection performance of this method was compared with those of the other methods reported in the literature, and were summarized in Table 1. For the detection range, except the methods of electrochemical, colorimetric, and optical fiber aptasensor, this method was superior to the other methods. Regarding the LOD, the method was only inferior to those of the electrochemical and the FRET. Furthermore, this method has some advantages of operation such as being simple to operate, low cost, and portable, which is suitable for fast on-site testing.

### 3.5. Selectivity

**Figure 5:**
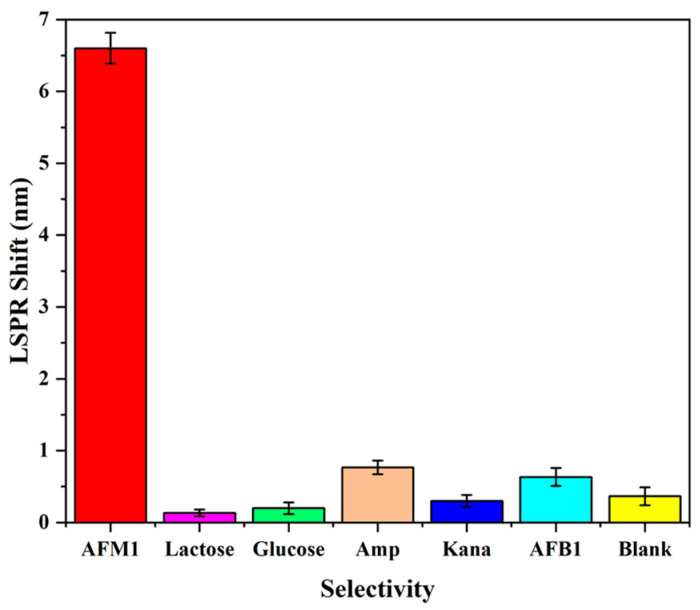
The selectivity of the fabricated LSPR biosensor.

Selectivity is an important performance parameter of analytical methods. The selectivity of this LSPR biosensor was investigated by the detection of 50 ng/mL interferents, including lactose, glucose, ampicillin, kanamycin, and AFB1. As shown in Figure 5, there were no significant LSPR peak shifts for the interferents and the buffer employed as a blank control; obviously, the LSPR peak has a significant shift for AFM1, which demonstrated that the biosensor has good selectivity for AFM1.

### 3.6. Repeatability and Reproducibility

**Figure 6:**
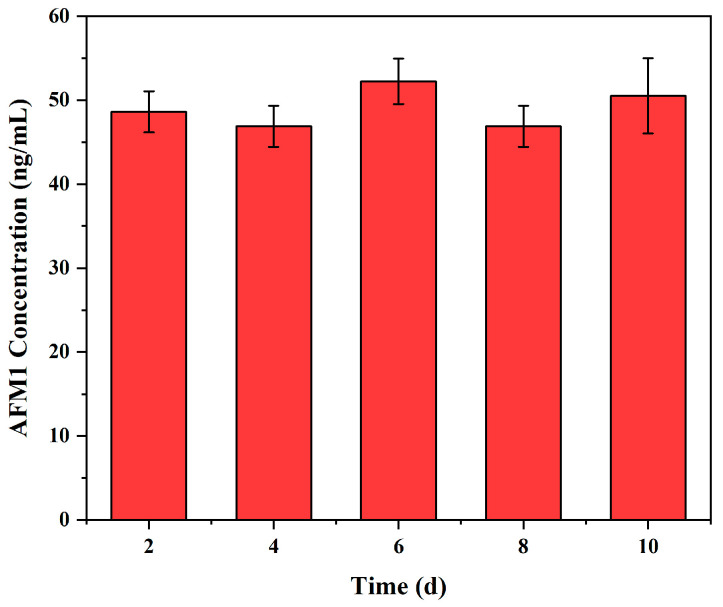
The reproducibility of the fabricated LSPR biosensor for AFM1 detection.

To assess the repeatability of the method, a 50 ng/mL AFM1 solution was tested 10 times on the same day under the identical conditions; the relative standard deviation (RSD) was 3.24%, demonstrating the method had good repeatability. To investigate the reproducibility of the method, each 50 ng/mL AFM1 solution was measured three times per 2 days. The results are summarized in Figure 6: each result was near 50 ng/mL. It demonstrated that the method had high reproducibility for AFM1 detection.

### 3.7. Performance Evaluation of the AuNP Multimers

**Figure 7:**
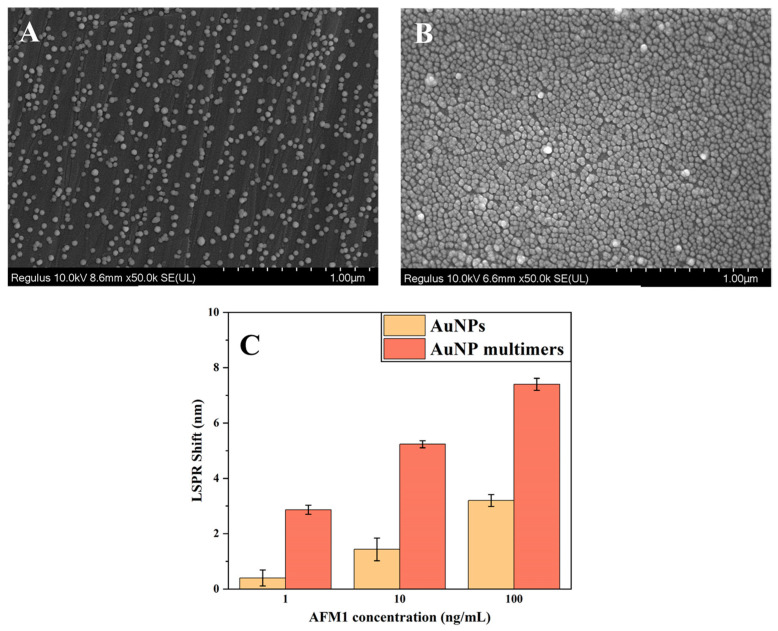
Performance evaluation of the AuNP multimers: (**A**) the SEM of the end face of a common FO LSPR sensor, (**B**) the SEM of the end face of the developed FO LSPR sensor, and (**C**) the comparison of the responses of the FO LSPR biosensors constructed with AuNPs and the AuNP multimers to 3 different concentrations of AFM1.

The SEM image of the AuNPs at the end face of the common LSPR sensor is shown in Figure 7A. The AuNPs were randomly distributed with large spacing, and no multimers were found.

For the FO LSPR sensor developed in this work, the SEM image of its end face is shown in Figure 7B. The density of the AuNPs increased, the multimers were obviously observed, and the spacing between the AuNPs was small.

Concentrations of 1, 10, and 100 ng/mL AFM1 were measured by the two kinds of the LSPR sensors. The results are shown in Figure 7C, in which the AuNP multimers sensor had a larger LSPR peak shift, the reason was that the hot spots generated by the nanogap in the constructed AuNP multimers improved the signal strength, resulting in the high AFM1 sensitivity of the developed FO LSPR biosensor.

### 3.8. Detection of Real Samples

One milk sample and three spiked samples (5 ng/mL, 10 ng/mL, and 50 ng/mL) were used to validate the detection performance of this method, and a reference method was used for the performance comparison. The results were presented in Table 2.

The recovery range of this method was from 88.9% to 118.4%, and the RSD range was 5.02–6.67%; they were acceptable. In addition, Student’s *t*-test was performed to compare the results of both methods, *p* > 0.05 indicated that the results of the two methods were not significantly different; therefore, the established method could be used for the detection of AFM1 for the real samples.

## 4. Conclusions

In this study, a FO LSPR biosensor utilizing the hot-spot effect of the AuNP multimers for the detection of AFM1 was successfully developed, and the biosensor exhibited high sensitivity, selectivity, and stability in AFM1 detection.

With the continuous progress of micro-nano processing technology, the development of FO LSPR biosensors in the future should prioritize the fabrication of two-dimensional metal nano-arrays, similar to AuNP multimers. By adjusting the periodicity of these arrays, the distribution of the hot spot can be significantly improved, thereby enhancing the strength of the electromagnetic field, which will improve the sensitivity of the sensors. Furthermore, with the development of intelligent instrumentation, the combination of FO LSPR biosensors and intelligent equipment will have great practical application prospects.

## Figures and Tables

**Table 1:** Comparison of the detection performance between this method and other sensing methods.

Methods	Detection Range (ng/mL)	LOD (ng/mL)	References
Fluorescence resonance energy transfer (FRET)	0.005–0.15	0.0015	[38]
Aptamer-based fluorescence	1–100	0.5	[39]
Immunofluorescence	0.2–0.8	0.07	[40]
Electrochemical	0.01–1000	0.037	[41]
Colorimetric	0.5–500.0	0.5	[42]
Plasmonic immunoassay	0.25–10	0.11	[43]
ELISA	0.1–0.6	0.05	[44]
Optical fiber aptasensor	1–1000	21	[45]
This method	0.05–100	0.04	This work

**Table 2:** The detection results of milk samples by this method and the reference method.

Spiked (ng/mL)	ELISA Method (ng/mL)	This Method (ng/mL)	RSD (%) **	Recovery (%)	*p* ***
0	N.D. *	N.D.	-	-	-
5	5.61 ± 0.12	4.46 ± 0.22	5.03	89.2	0.756
10	11.81 ± 0.06	11.84 ± 0.66	5.02	118.4	0.932
50	51.52 ± 0.14	44.45 ± 2.97	6.67	88.9	0.081

* N.D.—not detected. ** RSD—(Standard deviation)/mean × 100%, n = 3. *** *p* > 0.05 indicates no significant difference.

## Data Availability

Data will be made available on request.
